# The use of fully immersive virtual reality for screening neurodegenerative diseases: A systematic review of behavioral and diagnostic outcomes

**DOI:** 10.1002/dad2.70244

**Published:** 2026-01-07

**Authors:** Zhao Liu, Daniele Soria, Daniel Jie Lai, Jinbao Zhang, Sukhi Shergill, Chee Siang Ang

**Affiliations:** ^1^ School of Computing University of Kent Canterbury UK; ^2^ School of Sciences, Psychology, Arts and Humanities, Computing, Engineering, and Sport Canterbury Christ Church University Canterbury UK; ^3^ National Institute of Health and Care Research Applied Research Collaboration Kent Surrey and Sussex, Mill View Site Hove UK; ^4^ Care and Outcomes Research Centre University of Kent Canterbury UK; ^5^ Department of Psychosis Studies Institute of Psychiatry Psychology Neuroscience King's College London, Strand London UK; ^6^ Kent and Medway Medical School University of Kent Canterbury UK; ^7^ National Heart & Lung Institute Faculty of Medicine Imperial College London South Kensington Campus London UK

**Keywords:** Alzheimer's disease, cognitive screening, ecological validity, immersive virtual reality, machine learning, mild cognitive impairment, neuropsychology

## Abstract

Early detection of Alzheimer's disease (AD), Parkinson's disease (PD), and mild cognitive impairment (MCI) is crucial for timely intervention. Traditional cognitive screening tools lack ecological validity and sensitivity. Virtual reality (VR) provides realistic, controlled environments for assessing multidimensional cognition. This systematic review evaluated the diagnostic accuracy, feasibility, and applicability of immersive VR assessments for neurodegenerative screening. We searched PubMed, PsycINFO, and Embase for studies published June 2005 to April 2024. Eligible studies used head‐mounted displays in adults with MCI, early AD/PD, or dementia. Ten studies (*n* = 472) met criteria. Tasks targeted spatial memory, executive function, attention, and navigation. Several reported strong discriminations (area under the curve up to 0.89) and, when combined with machine learning, accuracies of 87% to 100%. Immersive VR shows promise as an ecologically valid, engaging, and scalable screening approach; however, standardization of tasks and outcomes, real‐world validation, and robust longitudinal evidence are needed to support clinical adoption.

**Highlights:**

- This review systematically describes the application of fully immersive virtual reality (VR) in the early screening of neurodegenerative diseases, with a focus on studies using head‐mounted devices to simulate real‐life tasks.
- Task types such as spatial memory, daily living simulations, and executive function assessments have demonstrated high sensitivity and specificity in diagnosing mild cognitive impairment (MCI) and early‐stage Alzheimer's disease (AD).
- Approximately one third of studies combined machine learning techniques to analyze multimodal behavioral data (e.g., path deviations, task duration, and language responses), significantly improving diagnostic accuracy.
- This study highlights methodological heterogeneity, small sample sizes, and the lack of longitudinal studies as current research limitations, and calls for future standardized, multicenter, and long‐term follow‐up studies to validate the predictive validity and real‐world applicability of VR tools.

## 1 INTRODUCTION

With the global population aging rapidly, neurodegenerative diseases (NDs), such as Alzheimer's disease (AD) and Parkinson's disease (PD), have emerged as some of the most pressing public health challenges of our time.^1^, ^2^ These disorders are characterized by a progressive deterioration of the structure and function of the nervous system, leading to a gradual decline in cognitive, behavioral, and motor abilities.^3^ Beyond the profound physical and psychological toll on individuals, NDs also impose substantial caregiving demands and financial burdens on families and health‐care systems.^4^ It is estimated that > 55 million people worldwide are currently living with dementia, with nearly 10 million new cases diagnosed each year.^5^ In the United Kingdom alone, the economic burden of dementia reached £34.7 billion in 2020, a figure expected to rise dramatically in the coming decades due to demographic shifts and increasing demand for long‐term care services.^6^

Timely identification of neurodegenerative diseases is critical for implementing early interventions and optimizing clinical outcomes. However, conventional screening methods, such as the Mini‐Mental State Examination (MMSE) and the Montreal Cognitive Assessment (MoCA), present notable limitations.^7^ Although these tools are widely used for their simplicity and clinical utility, they rely primarily on paper‐and‐pencil formats that may fail to capture the complexities of cognitive functioning in real‐world environments. Moreover, these tools often lack sensitivity to early‐stage cognitive impairments and are susceptible to practice effects with repeated administration, potentially compromising diagnostic accuracy over time.^7^

To address these limitations, researchers and clinicians have increasingly turned to digital technologies to modernize cognitive assessment practice.^8^ An alternative approach is to use computerized tests as screening tools, such as Cogstate^9^ and the Cleveland Clinic Cognitive Battery (C3B).^10^ They not only include enhanced measurement precision and self‐administration, but also cost effectiveness and reduced examiner bias.^11^ Moreover, mobile applications and tablet‐based assessments have gained traction in both clinical and community settings.^12^ However, compared to performance on traditional neuropsychological testing (e.g., Wisconsin Card Sorting Test, Stroop Test), there is a weak correspondence with activities of daily living.^13^ A more ecologically meaningful neuropsychological testing approach involves constructing a function‐led assessment.^14^ thereby enabling direct observation of everyday behavior and facilitating the tracing of underlying neuropsychological mechanisms. Conducting such assessments in naturalistic settings, however, is expensive, time consuming, logistically complex, and difficult to standardize.^15^

Virtual reality (VR) offers a promising solution to these requirements. Basically, the VR system can be classified into three levels: non‐immersive (e.g., computer displays), semi‐immersive (e.g., projection screens), and fully immersive.^16^ Full‐immersive VR (FIVR) encompasses immersion, interaction, and visual realism through head‐mounted displays (HMDs), surround sound, and other input devices.^17^ FIVR can also evaluate participants’ clinical, emotional, and social processing abilities in real time, and their approach is more closely aligned with real‐world functional performance.^18^ This enables cognitive testing tasks to simulate real‐life daily activities for assessment purposes,^19^ particularly in individuals with mild cognitive impairment (MCI) or early‐stage ND.^20^ Crucially, this immersion allows FIVR to uniquely target the cognitive domains most vulnerable in early neurodegeneration, such as complex executive functions and spatial navigation, which are often poorly captured by static 2D tests. For instance, the HMD‐based six‐domain battery (Cognitive Assessment by Virtual Reality [CAVIRE‐2]) effectively distinguishes MCI patients from cognitively healthy adults (area under the curve [AUC] = 0.88; sensitivity = 88.9%; specificity = 70.5%), providing cutoff values stratified by age/education level.^21^ Moreover, by casting tasks as instrumental activities of daily living (IADL), FIVR can simulate complex, lifelike settings while allowing for precise control over experimental variables and automatic data capture. Tasks such as navigating a virtual supermarket (e.g., VStore^22^), crossing a street,^23^ or performing a series of routine household actions,^24^ MCI patients wearing HMDs completed a point‐of‐interest (POI) task by physically walking within a virtual environment. The result was AUC = 0.90 for distinguishing “high/low AD risk MCI,” significantly outperforming the AUC = 0.57 of the best paper‐and‐pencil test.^25^

The gamified elements were incorporated into the task, enhancing user engagement and motivation and reducing the anxiety associated with it.^26^ These platforms can also capture rich behavioral data such as reaction times, eye movements, movement patterns, and task strategies, providing a multidimensional view of cognitive functioning.^27^ Recent studies have demonstrated the potential of VR‐based assessments to differentiate between healthy controls and individuals with cognitive impairment, such as the Cognition Assessment in Virtual Reality (CAVIR)^28^ and the RE@CH Assessment Module.^8^ Preliminary evidence also indicates strong user engagement with interventions among MCI patients and suggests that performance in VR tasks is sensitive to cognitive status.^26^

However, the clinical utility of FIVR is moderated by critical user‐level factors. Performance can be significantly influenced by age, education, and digital literacy, which may increase false positives or mask early deficiencies.^29^ For instance, older adults with lower digital familiarity may experience anxiety or difficulty with controllers, affecting task outcomes independent of cognitive status.^30^ Additionally, sensory limitations and susceptibility to cybersickness vary widely among older populations and are exacerbated by hardware heterogeneity.^29^ These factors underscore the need to evaluate not only diagnostic accuracy but also the feasibility and usability of these systems across diverse geriatric populations.

A growing body of review literature has explored the application of VR technology in cognitive research. Still, most studies have confused the levels of immersion and focused on intervention effects rather than assessment accuracy. For example, recent reviews on memory or spatial navigation have mixed different VR modes, failed to limit studies to HMD tasks, and often categorized patients by a single diagnosis (e.g., AD) rather than encompassing a holistic research of early neurodegenerative diseases such as MCI, AD, PD, and frontotemporal dementia (FD).^30^, ^31^ In contrast, this study strictly limited the inclusion of fully immersive HMD assessment tasks and categorized patients by early cognitive impairment domains (e.g., navigation/path integration, executive control, attention, and social cognition), extracting diagnostic and psychometric indicators to clarify clinical applicability—a focus largely absent in previous research. This review addressed a critical gap by synthesizing evidence on the diagnostic applications and implementation potential of such systems for early neurodegenerative screening. Specifically, the objectives of this review are: (1) to evaluate the diagnostic effectiveness of FIVR‐based cognitive assessments in detecting impairments associated with AD and PD; and (2) to assess the feasibility, usability, and clinical utility of FIVR systems in supporting health‐care professionals’ diagnostic processes. Through this analysis, we aim to evaluate the current state of FIVR technology in cognitive diagnostics and its potential to transform neuropsychological assessment in the era of digital health care.

## 2 MATERIALS AND METHODS

### 2.1 Search strategy

The following databases were searched: PubMed, PsycINFO, and Embase. Reference lists of key articles were also screened to identify additional eligible studies. Given the rapid development of VR‐based assessments, studies dated before June 22, 2005 were considered outdated and therefore excluded. Only studies published between June 22, 2005 and April 8, 2024 were included. No language restrictions were applied. June 2005 was selected as a pivotal juncture, coinciding with the release of widely adopted game engines such as Unity,^32^ which fundamentally transformed the research landscape by lowering development barriers. This timing validates the transition from high‐end visual simulation to accessible VR technology, a convergence trend that Zyda^33^ identified as the genesis of modern immersive applications.

The protocol for this review was not registered prospectively. However, the review followed the Preferred Reporting Items for Systematic Reviews and Meta‐Analyses (PRISMA) 2020 guidelines.

The Population/Intervention/Comparison/Outcome (PICO) framework was used to guide the development of search terms; however, the Population/Intervention/Outcome (PIO) model was adopted for this review as no restrictions were placed on the study design. The comparator component was excluded from the search strategy to maximize article retrieval. The search terms were structured as follows:

- Population (P): individuals with NDs.
- Intervention (I): fully immersive VR‐based assessment.
- Outcome (O): Task performance metrics (e.g., accuracy, response time), results of standard cognitive assessments (e.g., MMSE, MoCA), and diagnostic validity indicators such as AUC, classifier accuracy and where applicable, sensitivity and specificity.

The details of the search strategy were: (Neurodegenerative diseases* OR “neurodegenerative diseases”[MeSH]OR mild cognitive impairment OR dementia OR Alzheimer OR Parkinson OR Huntington OR Amyotrophic lateral sclerosis OR motor neuron OR older) AND (cognitive assessment OR cognition testing OR cognition screening) AND (Game OR Gamification OR virtual reality OR immersive OR head‐mounted display).

### 2.2 Study inclusion and exclusion criteria

Eligible studies included: (1) Patients with a confirmed diagnosis of MCI or dementia who were aged ≥ 60 years (aligned with the World Health Organization definition^34^); healthy control groups of any adult age >18 were accepted for comparative analysis. Diagnostic confirmation was required using standard clinical guidelines (e.g., DSM‐5) or validated neuropsychological assessments (e.g., MMSE, MoCA). (2) Interventions that were a fully immersive test run using VR HMDs. The specific task could be a cognitive test or a simulated work situation in everyday life. (3) Results of the experiment focused on a diagnosis or screening of the patient's cognitive state/condition. (4) Full text published in peer‐reviewed journals.

Studies were excluded if (1) they only included a healthy participant group, (2) the age range and type of disease did not match, (3) they were qualitative studies only assessing user acceptability without cognitive evaluation, or (4) they were systematic reviews and meta‐analyses, conference abstracts, and study protocols.

### 2.3 Selection process

All retrieved records were imported into Rayyan,^35^ a web‐based systematic review platform. Duplicate records were automatically detected and resolved within Rayyan. The platform was subsequently used to facilitate blinded title/abstract screening and full‐text review by the independent reviewers.

During the title and abstract screening stage, the lead author (Zhao Liu) conducted a preliminary screening of all records, and two independent reviewers (Daniel Jie Lai and Jinbao Zhang) each randomly assessed 50% of the studies for cross‐validation. Agreement with Daniel Jie Lai was moderate (Cohen Kappa = 0.24, prevalence‐adjusted and bias‐adjusted kappa [PABAK] = 0.88, observed agreement = 93.9%), while the agreement with Jinbao Zhang was high (Kappa = 0.49, PABAK = 0.88, observed agreement = 94.2%).

The discrepancy between the relatively low Kappa values and the high observed agreement can be attributed to two main factors. First, statistically, this reflects the well‐known “Kappa paradox”.^36^ Second, in practice, discrepancies often arose from ambiguities in study abstracts, particularly regarding VR hardware (e.g., distinguishing HMDs from non‐immersive screens).

During the full‐text screening stage, Zhao Liu and Daniel Jie Lai independently reviewed six full‐text articles and demonstrated perfect agreement (Kappa = 1.00, observed agreement = 100%). Zhao Liu and Jinbao Zhang reviewed 17 full‐text articles and achieved substantial agreement (Kappa = 0.74, observed agreement = 88.2%). Two disagreements were resolved through discussion and subsequently included. All discrepancies were addressed through reviewer discussion.

### 2.4 Data extraction

The following information was extracted from each included study:

1. Study characteristics, including author and year of publication, country, study design, participant characteristics, sample size (including presence or absence of a control group), and study duration. We extracted data as reported in the primary studies. When specific quantitative metrics (e.g., AUC) were missing, we reported available statistical outcomes (e.g., *p* values) and noted this limitation in the results table.
2. VR intervention characteristics, including the intervention description, type of VR intervention (e.g., assessment based, training based), and delivery format (e.g., VR devices, supporting systems).
3. To ensure a standardized synthesis of the heterogeneous outcomes, we adopted Bowen et al.’s framework for feasibility studies.^37^ Data were mapped into four key domains:
4. Acceptability: How the intended recipients react to the intervention. We extracted data on tolerability (e.g., simulator sickness, adverse events), dropout rates due to discomfort, and user experience feedback.
5. Practicality: The extent to which the intervention can be delivered when resources are constrained. We extracted metrics on administration time, technical issues (e.g., software bugs, hardware failures), and ease of use.
6. Implementation: The extent to which the intervention can be successfully delivered to the target population. We extracted data on completion rates and adherence to the study protocol.
7. Limited efficacy: The promise of the intervention being successful with the intended population. We extracted diagnostic performance metrics, including sensitivity, specificity, AUC, and correlations with standard neuropsychological tests (e.g., MMSE, MoCA).

Data extraction was independently conducted by three reviewers (Zhao Liu, Daniel Jie Lai, and Jinbao Zhang). No automation tools were used for data extraction. Any discrepancies were resolved through discussion.

### 2.5 Data synthesis and analysis

Given the heterogeneity of the included studies, a quantitative meta‐analysis was not feasible. Therefore, we conducted a narrative synthesis structured around the Bowen et al.^37^ framework outlined in section .2.4 Findings were systematically grouped into feasibility (implementation, practicality), acceptability, and diagnostic efficacy (limited efficacy) to facilitate cross‐study comparison. Because of the qualitative nature of the meta‐analysis, the effect indicators are presented using descriptive statistics from the original studies, including AUC, sensitivity, specificity, correlation coefficient (*r*), and *p* values for intergroup comparisons.

### 2.6 Heterogeneity and bias assessment

Sources of heterogeneity (e.g., differences in VR hardware and control group selection) were qualitatively assessed by comparing study characteristics and outcomes. Sensitivity analysis was not performed because this review uses descriptive integration. Similarly, due to the limited number of included studies (*n* < 10 for any specific outcome measure), the risk of bias due to missing results (publication bias) was not formally assessed using methods such as funnel plots; this practice is in accordance with Cochrane guidelines.

### 2.7 Risk of bias assessment

We assessed the methodological quality of the included studies using the QUADAS‐2 tool (Quality Assessment of Diagnostic Accuracy Studies, version 2),^38^ which is specifically designed to evaluate the risk of bias in diagnostic accuracy research. The tool comprises four domains: (1) patient selection, (2) index test, (3) reference standard, and (4) flow and timing. Each domain is assessed for risk of bias, while the first three domains are additionally evaluated for concerns regarding applicability. Assessment was performed independently by two reviewers (Zhao Liu and Daniele Soria), with any discrepancies resolved through discussion.

## 3 RESULTS

**FIGURE 1:**
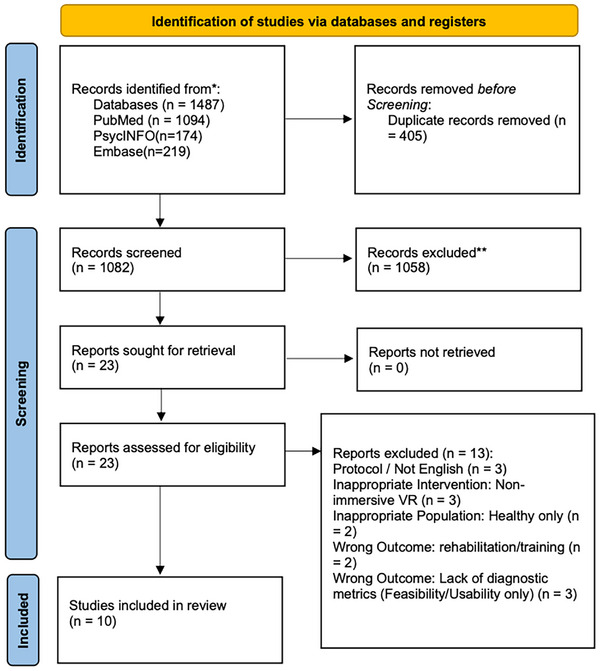
Flowchart of study selection based on Preferred Reporting Items for Systematic Reviews and Meta‐Analyses (PRISMA) guidelines. VR, virtual reality.

Initial database searches identified a total of 1487 articles. After the removal of 405 duplicates, 1082 articles were retained for title and abstract screening. Based on predefined exclusion criteria, 1059 articles were subsequently excluded, leaving 23 studies eligible for full‐text assessment. Upon detailed review, 10 articles met the inclusion criteria and were included in the current systematic review. A PRISMA flow diagram illustrating the detailed process of identification, screening, and inclusion of eligible studies is presented in Figure 1.

### 3.1 Study characteristics

This systematic review included 10 studies published between 2015 and 2023, conducted across multiple geographic regions, including Europe (UK, Spain), East Asia (China, Korea), North America (Canada), and South America (Brazil), see Table 1. A total of 472 participants were included, with individual sample sizes ranging from 12 to 108. The populations studied primarily consisted of individuals diagnosed with MCI, AD, or other early‐stage neurodegenerative conditions. Study designs varied across the included studies, with 40% (4/10) being experimental studies,^39^, ^40^, ^41^, ^42^ 30% (3/10) comparative studies,^20^, ^43^, ^44^ two feasibility studies,^45^, ^46^ and one validation study.^39^ One of these studies additionally incorporated machine learning methods for predictive modelling.^41^ The VR‐based assessments evaluated a range of cognitive domains, including spatial orientation, object‐location memory, executive function, language processing, and reaction time. VR hardware predominantly involved commercially available immersive devices such as HTC Vive and Oculus Quest, and only one assessment reported that the duration was 50 minutes. All studies used FIVR systems to assess spatial memory, executive functions, or other cognitive domains associated with early neurodegenerative decline. The summaries of all studies are in Table 1.

**TABLE 1:** Summary of studies using immersive VR for cognitive assessment of MCI and early AD.

Author, year	Country	Study design	Population	Sample/control	Duration	Intervention	Type	Delivery format	Outcome measures	Main findings
Castegnaro et al., 2022^39^	UK	Exp	aMCI vs. CG	23/24	50 min	Object‐location memory task (OLT)	Ass	iVR (HTC Vive)	Spatial binding accuracy, AUC	aMCI showed impaired accuracy (*p* < 0.001); AUC = 0.89 outperformed standard tests.
Wu et al., 2023^42^	China	Exp	MCI vs. CG	44/42	N/A	VR speech task + EEG	Ass	Oculus Quest 2 + Muse 2	Multimodal data: speech, EEG, digital scores	SVM (RBF) accuracy 87%, voting fusion reached 89.8%.
Moussavi et al., 2022^43^	Canada	Comp	AD/MCI/Others	65/20/8	N/A	Spatial orientation task	Ass	Wheelchair‐based VR	Spatial task scores	Differentiated AD and MCI (*p* < 0.001); effective clinical screening.
Da Costa et al., 2022^47^	Brazil	Vali	MCI vs. CG	19/29	N/A	SOIVET Maze and Route	Ass	Immersive VR maze	Accuracy, ROC	Moderate diagnostic accuracy; validated feasibility.
Campo‐Prieto et al., 2023^45^	Spain	Feas	PD	26	N/A	VR reaction time test	Ass	Immersive VR + TUG	RT, MMSE, TUG‐Cognitive	RT negatively correlated with MMSE (*p* = –0.576); useful fall risk indicator.
Park, 2022^44^	South Korea	Comp	MCI vs. CG	36/56	N/A	SCT‐VR spatial memory task	Ass	VR headset	SCT‐VR, MoCA‐K	SCT‐VR: sensitivity 0.944, specificity 0.964; test–retest ICC = 0.982.
Jang et al., 2023^46^	South Korea	Feas	MCI vs. CG	12/108	N/A	VR daily life simulation	Ass	Custom VR	VR score, MoCA, DHEA	AUC = 0.765; VR outperformed MoCA.
Serino et al., 2015^40^	Italy	Exp	aMCI/AD/CG	13/14/14	3 trials	Spatial representation in VR	Ass	Custom immersive VR	Allocentric/ errors	aMCI had allocentric deficits; AD had integrative spatial impairment.
Bayahya et al., 2021^20^	Saudi Arabia	Comp	Dem/MCI/CG	30/20/65	N/A	Smart 3D VR cognitive test	Ass	3D VR simulator	Mini‐Cog, memory, visuospatial	Kappa agreement up to 100%; VR valid as screening tool.
Tsai et al., 2021^41^	Taiwan, China	Exp	MCI/early AD vs. CG	6/6	N/A	VR supermarket + ML	Ass	HTC Vive VR + path tracking	46 behavioral & task indices	45/46 features significant; ML classifiers (e.g., SVM, XGBoost) accuracy = 100%.

Abbreviations: AD, Alzheimer's disease; aMCI, amnestic mild cognitive impairment; Ass, assessment; AUC, area under the curve; CG, control group; Comp, comparative; Dem, dementia; DHEA, dehydroepiandrosterone; EEG, electroencephalogram; Exp, experimental; Feas, feasibility; ICC, intra‐class correlation; iVR, immersive virtual reality; MCI, mild cognitive impairment; ML, machine learning; MMSE, Mini‐Mental State Examination; MoCA, Montreal Cognitive Assessment; PD, Parkinson's disease; ROC, receiver operating characteristic; RT, reaction time; SCT‐VR, social cognition training with virtual reality; SOIVET, Spatial Orientation in an Immersive Virtual Environment Test; SVM, support vector machine; TUG, Timed Up and Go; Vali, validation; VR, virtual reality.

### 3.2 Feasibility and acceptability

A detailed feasibility and acceptability structure based on Bowen et al.’s^37^ framework is presented in Table 2.

- Acceptability: The interventions were generally well tolerated, and participants reported high engagement, particularly in tasks with high ecological validity, such as the virtual supermarket.^20^, ^41^ Although some studies reported minimal adverse effects (e.g., Campo‐Prieto et al.^45^), others noted dropout due to motion sickness, particularly in MCI groups (e.g., Da Costa et al.^47^).
- Implementation: Most studies demonstrated high feasibility. Completion rates were generally high across most studies with explicit rates > 90% in Castegnaro et al.^39^ and Tsai et al.^41^ and the full adherence to the task was reported by Campo‐Prieto et al.^45^ However, Moussavi et al.^43^ reported that longitudinal retention was low (30%).
- Practicality: Most systems used commercially available headsets (e.g., HTC Vive, Oculus Quest), enabling practical deployment. A few studies didn't specify the assessment time, and most would take 5 to 20 minutes to complete the testing.
- Limited efficacy: The preliminary efficacy of these VR tools for diagnostic screening—including sensitivity, specificity, and discriminatory power—is detailed in section 3.3 and summarized in Table 3.

**TABLE 2:** Assessment of feasibility and acceptability based on Bowen et al.’s framework.

Author, year	Acceptability (e.g., tolerance, satisfaction)	Practicality (e.g., time, tech issues)	Implementation (e.g., completion, attrition)
Castegnaro et al., 2022^39^	No fatigue or motion sickness reported.	Total time ≈ 50 minutes (including set‐up/practice).	100% completion (*n* =100).
Wu et al., 2023^42^	Not reported.	Used wearable EEG (MUSE 2) + Oculus Quest 2.	86 participants completed.
Moussavi et al., 2022^43^	Wheelchair set‐up reduced kinetosis; simple to use.	Quick administration (5–10 minutes).	Longitudinal retention low (30%), but cross‐sectional *n* = 93 analyzed.
Costa et al. 2021^47^	Dropout rate: 11.4% (controls) and 14.8% (MCI) due to cybersickness.	Task shortened (18 turns) to reduce anxiety/difficulty.	8 participants dropped out (total *n* = 48 analyzed).
Campo‐Prieto et al. 2023^45^	No adverse effects; 0 SSQ symptoms.	Short duration (60s test trials).	100% completion (*n* = 26).
Park 2022^44^	Not reported.	Controller‐based HMD set‐up.	92 participants completed.
Jang et al. 2023^46^	VRSQ scores indicated no severe sickness (mean ≈ 15–19).	Average time 18.9 minutes. 30 participants excluded due to software error.	120/150 completed (80% retention after tech exclusions).
Serino et al. 2015^40^	1 aMCI patient failed to complete tasks (reason unspecified).	NeuroVirtual 3D platform used.	44/45 completed.
Bayahya et al. 2021^20^	No withdrawal; reported as “user friendly.”	Time < 5 minutes.	100% completion (*n* = 115).
Tsai et al. 2021^41^	No additional burden reported; enhanced immersion.	High‐end PC required (RTX 2070) for rendering.	100% completion (small sample *n* = 12).

*Note*: The “limited‐efficacy” domain of Bowen et al.’s framework, which corresponds to diagnostic performance, is presented separately in Table 3 to allow for a detailed integration with quality assessment.

Abbreviations: aMCI, amnestic mild cognitive impairment; EEG, electroencephalogram; HMD, head‐mounted device; MCI, mild cognitive impairment; SSQ, Simulator Sickness Questionnaire; VRSQ, Virtual Reality Sickness Questionnaire.

**TABLE 3:** Summary of diagnostic performance and quality assessment (QUADAS‐2).

Author, Year	Outcome measure	Comparison group	Diagnostic performance metrics	Patient selection	Index test	Reference standard	Flow and timing
Castegnaro et al., 2022^39^	Object‐location memory	aMCI vs. HC	AUC = 0.89	Low	Low	Low	Low
Wu et al., 2023^42^	Multimodal VR features	MCI vs. HC	Accuracy = 89.8%	Low	Low	Low	Unclear
Moussavi et al., 2022^43^	VR navigation	AD vs. MCI	Sig. diff. (*p* < 0.001)	Low	Unclear	Low	Low
Park, 2022^44^	SCT‐VR	MCI vs. HC	AUC = 0.99; Sens = 94%; Spec = 96%	Unclear	Unclear	Low	Low
Costa et al., 2021^47^	SOIVET Maze & Route	MCI vs. HC	Maze AUC = 0.73; route AUC = 0.70	High	Low	Low	High
Jang et al., 2023^46^	VARABOM	MCI vs. HC	AUC = 0.77; Sens = 83%; Spec = 72%	Unclear	Low	High	Unclear
Bayahya et al., 2021^20^	Smart health VR	Dementia vs. HC	Accuracy = 97.2%	Unclear	Unclear	Unclear	Unclear
Campo‐Prieto et al., 2023^45^	VR reaction time	Fallers vs. non‐fallers	AUC = 0.74; Sens = 70%; Spec = 75%	Low	Low	Low	Low
Tsai et al., 2021^41^	VR supermarket	MCI/AD vs. HC	Accuracy = 100%	High	Low	Unclear	Unclear
Serino et al., 2015^40^	VR spatial memory	aMCI vs. HC	Sig. diff. (*p* < 0.01)	Low	Unclear	Low	Low

Abbreviations: AD, Alzheimer's disease; aMCI, amnestic mild cognitive impairment; AUC, area under the curve; HC, healthy control; MCI, mild cognitive impairment; QUADAS‐2, Quality Assessment of Diagnostic Accuracy Studies, version 2; Risk of Bias ratings, high, low, unclear; SCT‐VR, social cognition training with virtual reality; Sens, sensitivity; Sig. diff., significant difference; SOIVET, Spatial Orientation in an Immersive Virtual Environment Test; Spec, specificity; VARABOM, virtual reality‐based mild cognitive impairment monitoring; VR, virtual reality.

### 3.3 Diagnostic performance and cognitive relevance

The diagnostic performance metrics and associated quality assessments (QUADAS‐2) for all included studies are summarized in Table 3.

### 3.4 Object localization and spatial memory tasks

Castegnaro et al.^39^ developed three immersive VR subtasks, such as object location memory, object recognition, and object‐context association, to evaluate object–space binding deficits in individuals with amnestic mild cognitive impairment (aMCI). The tasks were delivered via an HTC Vive platform and incorporated active navigation and multidimensional performance metrics (e.g., absolute distance error). Task performance was also correlated with cerebrospinal fluid biomarkers (e.g., tau), improving sensitivity to AD pathology. The object location memory task demonstrated superior discriminative accuracy (AUC = 0.89) compared to conventional tools, while the association of object context was significantly associated with the volume of the lateral entorhinal cortex, highlighting the utility of the task in the detection of preclinical AD.

Park^44^ introduced a VR‐based spatial memory reconstruction task (social cognition training with VR [SCT‐VR]) requiring participants to encode and reposition multiple objects within a virtual scene. Developed in Unity and executed on an HMD platform with controller‐based input, the task captured spatial reconstruction accuracy and time metrics. The system yielded exceptionally high classification accuracy between MCI and healthy older adults (sensitivity = 0.944; specificity = 0.964), outperforming standard instruments such as the MoCA and supporting the diagnostic utility of spatial memory tasks in early cognitive screening. However, the QUADAS‐2 assessment (Table 3) indicates an unclear risk of bias for Park due to insufficient details on patient selection.

Wu et al.^42^ designed a multimodal VR system that integrated voice‐based tasks with electroencephalogram (EEG) acquisition to assess executive and language function in MCI. Participants engaged in iterative question‐and‐answer and object‐matching tasks within a virtual environment, while EEG and behavioral data (e.g., response latency, speech accuracy) were simultaneously recorded. Multiple machine learning (ML) models, including support vector machine (SVM), Random Forest, and XGBoost, were evaluated using leave‐one‐out cross‐validation (LOOCV) to maximize robustness with a limited sample size. The SVM achieved the highest accuracy (AUC = 87%), and gamma‐band power positively correlated with cognitive load, demonstrating the potential of VR–EEG integration for neurocognitive profiling.

### 3.5 Spatial orientation and navigation tasks

Serino et al.^40^ implemented a spatial orientation conversion task using immersive VR to investigate allocentric‐to‐egocentric transformation impairments in AD and aMCI. Conducted in a cave automatic virtual environment simulating 3D indoor spaces, the task used passive navigation and measured spatial conversion accuracy and reaction time. AD and aMCI participants showed significantly reduced accuracy in allocentric‐to‐egocentric transformations compared to controls, supporting the “mental frame syncing” hypothesis and confirming the task's sensitivity to early spatial processing deficits.

Da Costa et al.^48^ developed the Spatial Orientation in an Immersive Virtual Environment Test (SOIVET) system, comprising two immersive tasks—maze navigation and route reproduction—to evaluate spatial learning impairments in MCI. The participants navigated autonomously using Oculus Rift headsets. Performance on both tasks correlated significantly with conventional spatial assessments (e.g., Mental Rotation Test) and demonstrated moderate discriminative capacity, underscoring the ecological value of VR‐based spatial testing.

Moussavi et al.^43^ created a spatial working memory task using HTC Vive in which participants navigated a complex virtual building and returned to a starting point. The outcome measures included the accuracy and time of the reconstruction of the path, reflecting spatial updating and the navigational strategy. Participants in MCI exhibited greater deviation and recall delay in the path than controls, with results significantly correlated with MoCA scores, validating the clinical utility of the task for early detection of spatial impairment.

### 3.6 Simulated activities of daily living and executive function tasks

Tsai et al.^41^ developed an immersive VR‐based supermarket task to assess spatial memory and multitasking in MCI. Implemented on Oculus Quest 2, the system involved navigation, item selection, and pricing, with controller input and voice feedback. A multimodal feature set—including task duration, error rates, and path efficiency—was modelled using SVM and XGBoost classifiers, achieving 100% accuracy in distinguishing MCI from controls. However, this perfect accuracy should be interpreted with caution, given the small sample size (*n* = 12) and high risk of bias in patient selection (Table 3).

Campo‐Prieto et al.^45^ introduced a VR‐based reaction wall task targeting dynamic attention and motor responsiveness in PD. Activated on Oculus Quest 2 with motion‐tracked input, the task recorded reaction time and strike accuracy in response to visual stimuli. Performance was moderately negatively correlated with MMSE scores (*ρ* = −0.576, *p* = 0.002) and positively with Timed Up and Go (TUG) scores, indicating both cognitive and motor predictive value.

Jang et al.^46^ created a functional VR scenario simulating home‐care routines to evaluate daily task execution in older adults. Tasks such as medication retrieval and caregiver calls were completed within HTC Vive environments, with real‐world object affordances and path tracing. MCI participants showed lower task adherence and operational sequencing compared to healthy controls. The results show that the sensitivity of simulated ADL‐based tasks to multidomain cognitive deficits.

Bayahya et al.^20^ designed the “Smart Supermarket” immersive VR system, integrating route guidance, object selection, and verbal interaction for dementia risk screening. Built in Unity, the system collected behavioral indicators such as navigation efficiency and repetitive errors. Using a Random Forest classifier, the model yielded high agreement with Mini‐Cog results (Kappa = 0.93), highlighting the task's dual advantage of ecological validity and diagnostic sensitivity.

### 3.7 Comparison with clinical tools and artificial intelligence integration

Across studies, 7 of 10 compared VR task performance with standard clinical tools such as the MMSE, MoCA, or Mini‐Cog, covering key cognitive domains such as memory, spatial ability, and executive function (see Table 4 for details). For example, Park^44^ and Tsai et al.^41^ demonstrated strong alignment with MoCA and MMSE, while Castegnaro et al.^39^ achieved an AUC of 0.89 for MoCA‐based differentiation. Da Costa et al.^49^ incorporated multiple tools (Addenbrooke's Cognitive Examination Revised, Benton Judgment of Line Orientation, Tower of London) to broaden domain coverage. Serino et al.^40^ and Jang et al.^46^ performed only partial comparisons, and Moussavi et al.^43^ did not apply clinical benchmarks. VR metrics generally showed moderate‐to‐strong correlations with these established instruments, while offering superior sensitivity for detecting subtle functional deficits (see Table 3).

**TABLE 4:** Comparison of VR tasks to standard clinical tools.

Author, Year	VR task	Compared to clinical tool	Tool(s)
Park, 2022^44^	Spatial memory	Yes	MoCA‐K
Tsai et al., 2021^41^	Virtual supermarket + ML	Yes	MoCA, MMSE
Bayahya et al., 2021^20^	VR navigation & memory	Yes	Mini‐Cog
Campo‐Prieto et al., 2023^45^	Reaction time	Yes	MMSE
Serino et al., 2015^40^	Allocentric‐egocentric spatial transformation	Partially	MMSE (group classification only)
Da Costa et al., 2022^49^	SOIVET Maze & Route	Yes	ACE‐R, BJLO, Tower of London
Castegnaro et al., 2022^39^	Object‐location memory	Yes	MoCA
Jang et al., 2023^46^	Grandchild care scenario	Partially	MoCA (AUC only)
Wu et al., 2023^42^	VR speech task + EEG	Yes	MoCA, MMSE
Moussavi et al., 2022^43^	Target localization in virtual building	No	None

Abbreviations: ACE‐R, Addenbrooke's Cognitive Examination Revised; AUC, area under the curve; BJLO, Benton Judgment of Line Orientation test; EEG, electroencephalogram; Mini‐Cog, brief cognitive screening tool combining memory and clock drawing; ML, machine learning; MMSE, Mini‐Mental State Examination; MoCA, Montreal Cognitive Assessment; MoCA‐K, Montreal Cognitive Assessment Korean version; SOIVET, Spatial Orientation in an Immersive Virtual Environment Test; Tower of London, neuropsychological test of planning ability; VR, virtual reality.

Notably, three studies integrated artificial intelligence (AI)–based classifiers to enhance diagnostic performance. Tsai and Wu used SVM and XGBoost for behavior–EEG fusion, achieving 100% and 87% classification accuracies, respectively. Bayahya et al. applied a Random Forest model that matched Mini‐Cog diagnoses (Kappa = 0.93).^20^, ^41^, ^42^ However, none of these studies reported external validation on independent datasets, relying instead on internal cross‐validation (e.g., LOOCV in Wu et al.,^42^ 10‐fold CV in Tsai et al.^41^).

### 3.8 Synthesis of evidence

In summary, a comprehensive analysis of existing evidence indicates that FIVR technology holds considerable potential for screening MCI and dementia, although research findings exhibit significant heterogeneity. Current trends suggest that VR tasks targeting key cognitive domains, such as spatial navigation and object‐location memory, generally demonstrate superior diagnostic performance compared to traditional paper‐and‐pencil tests, with multiple studies reporting AUC values consistently > 0.85. Under conditions of appropriate task duration and manageable interactive burden, most studies report high task completion rates and low incidence of virtual motion sickness, broadly supporting the technology's feasibility and acceptability. Furthermore, some studies used case–control sampling or failed to detail blinding procedures and measurement protocols, resulting in unclear risk of bias in QUADAS‐2 assessments. Substantial evidence gaps persist, particularly concerning the standardization of VR tasks and metrics, reporting of assessment duration, longitudinal retention rates, and the evaluation of real‐world applicability. These cross‐study patterns highlight both the potential of immersive VR technology in early cognitive screening and the fragmented developmental stage currently prevailing in this field. The risk of reporting bias (e.g., publication bias) could not be statistically assessed but remains a potential limitation, as studies with negative findings may be underrepresented.

## 4 DISCUSSION

This systematic review synthesized current evidence regarding the application of FIVR in screening and early diagnosis of NDs, focusing primarily on AD, MCI, and PD. The review specifically evaluated the diagnostic effectiveness and clinical feasibility of immersive VR systems, aiming to clarify their role in modern cognitive diagnostics and potential implications for clinical practice.

### 4.1 Diagnostic effectiveness of VR‐based cognitive assessments

The findings of this review suggest that immersive VR technologies offer considerable promise for the early and accurate detection of cognitive impairments associated with NDs. Across the included studies, VR‐based cognitive tasks demonstrated diagnostic accuracy that was not only comparable to, but in several cases surpassed, that of established neuropsychological tools such as the MMSE and the MoCA. However, rather than expecting FIVR to replace these traditional tools, our findings show that they can serve a complementary role. Although standard neuropsychological tests have excelled in quantifying specific cognitive performance, such as memory recall, VR tasks excel at capturing complex functional–behavioral data, such as navigational^22^, ^40^, ^43^, ^47^ and multitasking performance.^20^, ^41^, ^45^, ^46^ This ecological validity has profound implications for clinical decision making: compared to static paper‐and‐pencil test scores, patients’ performance in naturalistic simulation settings better predicts their capacity for independent living. This facilitates an earlier rehabilitation plan or the implementation of safety assessments.^48^

A key trend identified is the integration of ML to enhance diagnostic precision. For instance, Tsai et al.^41^ achieved 100% classification accuracy using SVM and XGBoost classifiers. Although promising, such results must be interpreted with significant caution. The application of complex ML algorithms to small datasets (*n* = 12 in Tsai et al.) can lead to a high risk of overfitting, in which models memorize noise rather than learning generalizable patterns.^50^ Furthermore, the lack of external validation in these studies limits their clinical translational value. Without validation of the model in independent cohorts, the reported “perfect” accuracy is likely to overestimate actual clinical diagnostic utility. Establishing transparent reporting standards for ML models such as the Transparent Reporting of a Multivariable Prediction Model for Individual Prognosis or Diagnosis (TRIPOD)^51^ is crucial, as it will propel the field from proof‐of‐concept to clinical practice.

Another feature of VR technology is its ability to replicate real‐world contexts while maintaining experimental control and allowing for repeated, low‐cost testing under consistent conditions. This addresses the long‐standing ecological validity limitations of traditional cognitive assessments. By embedding cognitive tasks within naturalistic environments, VR assessments can yield more detailed and representative behavioral data. Studies such as Kourtesis et al.^52^ and Romero‐Ayuso et al.^53^ have demonstrated the alignment between behavioral responses in VR environments and those observed in real‐world contexts. Others, such as Parsons^54^ have highlighted the objectivity and standardization of behavior‐based VR data capture.

Tasks embedded in VR environments, such as those conducted in a virtual supermarket^22^, ^41^ or a simulated home caregiving scenario^46^ have shown particular utility in identifying early cognitive decline. This ecological validity not only enhances diagnostic precision but may also improve participant engagement and reduce evaluation‐related stress, thereby contributing to the overall reliability of the screening process. Similar benefits have been reported in studies examining other clinical populations in which ecological demands are integral to real‐life functioning.^52^ Taken together, these findings underscore the promise of VR as a practical and innovative approach to the early detection of cognitive impairment related to neurodegeneration.

### 4.2 Feasibility, usability, and clinical implementation

The feasibility‐related outcomes reported across the studies consistently underscored the practicality and acceptability of VR‐based cognitive assessments. High task completion rates (> 90%) in several studies^39^, ^41^ suggested VR's ease of use and positive patient compliance. Usability and engagement were generally high, supported by intuitive interfaces and naturalistic task scenarios, facilitating immersive and meaningful interaction. The absence of significant adverse events in most studies further highlights VR's suitability for routine clinical deployment. However, mild adverse effects such as fatigue and cybersickness were reported in one study,^45^ emphasizing the need for careful task design, especially for populations with potential physical or sensory limitations.

The adoption of commercially available immersive devices (e.g., Oculus Quest, HTC Vive) represents another critical strength. These devices, being cost effective, easily deployable, and user friendly, significantly reduce barriers to clinical implementation. Additionally, their compatibility with advanced analytic techniques, such as ML, further broadens their applicability and enhances diagnostic precision through the analysis of complex behavioral patterns.

Nevertheless, obstacles to routine clinical application remain. First, digital literacy remains a significant moderating factor; while existing research shows high engagement, the frequent recruitment of enthusiastic volunteers may mask anxiety or resistance within a broader older population.^55^ Second, operating costs extend beyond hardware procurement, including software licensing, troubleshooting, and the need for dedicated physical space in clinics.^56^ Third, training clinicians is a critical bottleneck. Although VR can standardize data collection, the interpretation of these data and the integration into concrete clinical guidelines still rely heavily on expert clinical judgement^57^. Therefore, successful implementation necessitates not only user‐friendly software but also specialized data analysis capabilities.

### 4.3 Limitations of current evidence and recommendations for future research

Although the aggregated data support FIVR as a potential cognitive assessment, the evidence was constrained by limitations identified in the QUADAS‐2 assessment. A pervasive problem is the high risk of bias in patient selection, primarily stemming from the use of case–control study designs. By recruiting patients with a “clear diagnosis” of dementia and “healthy” controls, studies artificially amplify the diagnostic contrast, which may lead to spectrum bias. This may explain the near‐perfect AUC values (0.99–100)^41^, ^44^ reported in some studies, which can significantly decrease when applied to the “complex” heterogeneous populations commonly found in primary care.

Furthermore, the lack of clear blinding in several studies poses a risk of operational bias; if the operator is aware of the participant's diagnosis, they might subtly guide the participant during the VR task. Small sample sizes further weaken the statistical power and the reliability of accuracy estimates.

Methodologically, few studies incorporated multimodal physiological data (e.g., EEG and eye‐tracking), which could enhance diagnostic sensitivity. Participant‐level factors—such as age, education, and digital literacy—were also rarely considered, despite their potential influence on usability and performance, particularly in older adults. These methodological flaws suggest that while the potential of VR is high, the current evidence base is likely overly optimistic.

Although many studies aimed to enhance ecological validity through realistic virtual scenarios and comparisons with standardized cognitive tools, none directly compared VR performance to real‐world behavior. Similarly, no study systematically examined the effect of immersion level (e.g., HMDs vs. non‐immersive platforms) on cognitive outcomes. Furthermore, most studies relied on cognitive screening tools (e.g., MoCA) as the reference standard, rather than robust biological markers such as positron emission tomography, magnetic resonance imaging, or cerebrospinal fluid biomarkers, limiting the ability to validate VR outcomes against the underlying pathology.

### 4.4 Recommendations for future research

Future research should prioritize large‐scale, multicenter studies using standardized VR protocols to enable meaningful cross‐study comparisons and support robust evidence synthesis. Longitudinal designs are also critically needed to evaluate the predictive validity of VR‐based cognitive assessments in tracking clinical progression and forecasting disease trajectories over time.

In detail, to advance the field from exploratory to clinical adoption, future research should address the following directions:

- Standardization of protocols: There is an urgent need for a standardized “core outcome set” for VR cognitive trials, defining essential reporting metrics (e.g., hardware specs, locomotion methods, cybersickness scores) to facilitate meta‐analysis.
- Robust study designs: Future studies should move beyond case–control designs to consecutive cohort studies in primary care settings to determine the true predictive value of VR tools in undifferentiated populations.
- External and cross‐cultural validation: As VR relies heavily on visual cues, tools developed in one culture (e.g., a Western supermarket) may not translate to others. Cross‐cultural validation studies are necessary to ensure global applicability.
- Multimodal integration: Integrating VR with biomarkers (e.g., EEG, eye‐tracking) could uncover latent neurocognitive signatures, thereby improving sensitivity to preclinical stages (e.g., MCI) in which behavioral deficits are subtle.

## 5 SUMMARY AND CONCLUSIONS

In conclusion, this systematic review supports the effectiveness and clinical feasibility of immersive VR‐based cognitive assessments for the early detection of neurodegenerative disorders. By addressing limitations inherent in conventional cognitive testing approaches, immersive VR offers transformative potential in neuropsychological assessment, promising earlier diagnosis, targeted intervention, and improved patient outcomes. As technology and digital health‐care continue to evolve, VR is poised to become an integral component of cognitive diagnostic pathways, substantially shaping the future landscape of neuropsychological evaluation and patient management.

## CONFLICT OF INTEREST STATEMENT

The authors declare no conflicts of interest.

## Supporting information




Supporting Information

Supporting Information

Supporting Information

Supporting Information
