# Supplementary figures and images for: The use of fully immersive virtual reality for screening neurodegenerative diseases: A systematic review of behavioral and diagnostic outcomes
Source: Alzheimers Dement (Amst). 2026 Jan 7;18(1):e70244. doi: 10.1002/dad2.70244 (PMC12780346; doi:10.1002/dad2.70244)

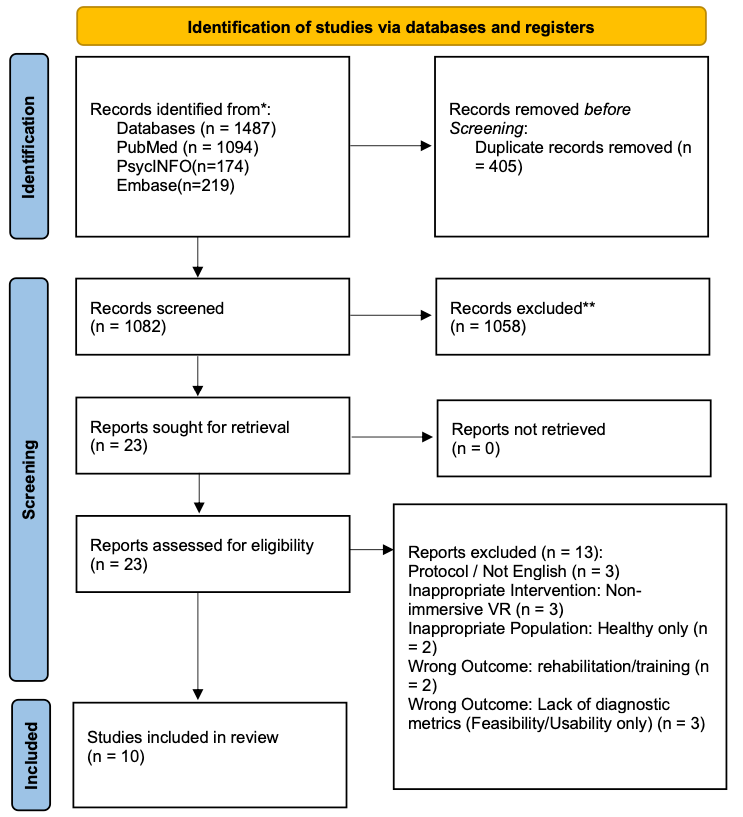

Supplement: Supplementary file 1 — Supporting Information [file DAD2-18-e70244-s003.tiff]
